# Phylogeny of Plant Calcium and Calmodulin-Dependent Protein Kinases (CCaMKs) and Functional Analyses of Tomato CCaMK in Disease Resistance

**DOI:** 10.3389/fpls.2015.01075

**Published:** 2015-12-08

**Authors:** Ji-Peng Wang, Jean-Pierre Munyampundu, You-Ping Xu, Xin-Zhong Cai

**Affiliations:** ^1^Institute of Biotechnology, College of Agriculture and Biotechnology, Zhejiang UniversityHangzhou, China; ^2^Centre of Analysis and Measurement, Zhejiang UniversityHangzhou, China; ^3^State Key Laboratory of Rice Biology, Zhejiang UniversityHangzhou, China

**Keywords:** calcium and calmodulin-dependent protein kinase (CCaMK), genome-wide identification, phylogeny, resistance, hydrogen peroxide (H_2_O_2_), tomato

## Abstract

Calcium and calmodulin-dependent protein kinase (*CCaMK*) is a member of calcium/calmodulin-dependent protein kinase superfamily and is essential to microbe- plant symbiosis. To date, the distribution of *CCaMK* gene in plants has not yet been completely understood, and its function in plant disease resistance remains unclear. In this study, we systemically identified the *CCaMK* genes in genomes of 44 plant species in Phytozome and analyzed the function of tomato *CCaMK* (*SlCCaMK*) in resistance to various pathogens. CCaMKs in 18 additional plant species were identified, yet the absence of *CCaMK* gene in green algae and cruciferous species was confirmed. Sequence analysis of full-length CCaMK proteins from 44 plant species demonstrated that plant *CCaMKs* are highly conserved across all domains. Most of the important regulatory amino acids are conserved throughout all sequences, with the only notable exception being observed in N-terminal autophosphorylation site corresponding to Ser 9 in the *Medicago truncatula CCaMK*. *CCaMK* gene structures are similar, mostly containing six introns with a phase profile of 200200 and the exception was only noticed at the first exons. Phylogenetic analysis demonstrated that CCaMK lineage is likely to have diverged early from a calcium-dependent protein kinase (CDPK) gene in the ancestor of all nonvascular plant species. The *SlCCaMK* gene was widely and differently responsive to diverse pathogenic stimuli. Furthermore, knock-down of *SlCCaMK* reduced tomato resistance to *Sclerotinia sclerotiorum* and *Pseudomonas syringae* pv. *tomato* (*Pst*) DC3000 and decreased H_2_O_2_ accumulation in response to *Pst* DC3000 inoculation. Our results reveal that *SlCCaMK* positively regulates disease resistance in tomato via promoting H_2_O_2_ accumulation. *SlCCaMK* is the first *CCaMK* gene proved to function in plant disease resistance.

## Introduction

Calcium and calmodulin (CaM)-dependent protein kinase (CCaMK) belongs to calcium/CaM-dependent protein kinase superfamily (Harmon et al., 2000). CCaMKs are plant-specific and have a well-documented function to decode and transduce the calcium spiking signals during rhizobial and arbuscular mycorrhizal (AM) symbioses in legumes (Gleason et al., 2006; Tirichine et al., 2006; Kosuta et al., 2008; Hayashi et al., 2010; Madsen et al., 2010). Importantly, non-legume CCaMKs displayed a similar function when transformed into legumes (Godfroy et al., 2006; Chen et al., 2007; Banba et al., 2008). Despite being plant specific, CCaMKs are distinct from plant calcium-dependent protein kinases (CDPKs) and other plant serine/threonine kinases. On the contrary, they are highly similar to animal CaM-dependent protein kinase II (CaMKII), carrying a serine/threonine kinase domain, an overlapping autoinhibitory/CaM-binding (CaMB) domain and three visinin-like EF-hand calcium binding motifs (Patil et al., 1995; Mitra et al., 2004). CCaMK activity requires both free Ca^2+^ and Ca^2+^ bound to CaM (Ca^2+^/CaM) with visinin-like EF-hand domain and CaMB domain acting as Ca^2+^-triggered switch and autophosphorylation-triggered molecular switch, respectively (Takezawa et al., 1996; Sathyanarayanan et al., 2000).

CCaMK is subject to both positive and negative autoregulation via conserved autophosphorylation sites, including one located in the Ser/Thr kinase domain and the others in the CaMB domain (Liao et al., 2012; Shimoda et al., 2012). In their established model illustrating Ca^2+^ and Ca^2+^/CaM-dependent regulation of CCaMKs, Singh and Parniske (2012) demonstrated that the conserved autophosphorylation site T265 of *Lotus japonicus* CCaMK is required for negative autoregulation of the kinase activity in the absence of Ca^2+^ by engaging in a hydrogen-bond network involving residues S237, K264, E313, and R317. The autoinhibition release and kinase activity is brought about by disruption of this hydrogen-bond network upon Ca^2+^ binding at EF-hand motifs, resulting in T265 autophosphorylation in the kinase domain and an increased affinity for CaM. The Ca^2+^/CaM complex binding to the autoinhibitory/CaMB domain induces conformational change that leads to high substrate phosphorylation activity of CCaMK. On the other hand, the Ca^2+^/CaM-dependent negative autoregulation of LjCCaMK is achieved via autophosphorylation at S337 site in the CaMB domain, impairing Ca^2+^/CaM binding. The LjCCaMK S337 autophosphorylation site allows CaM binding only in the unphosphorylated state and the authors underscored the necessity of Ca^2+^/CaM-dependent negative regulation of CCaMKs for intracellular infection. So far, two regulatory phosphorylation sites (T265 and S337) have been identified in LjCCaMK (Liao et al., 2012), while three sites (S9, T271, and S344) have been reported in *Medicago truncatula* CCaMK and other phosphorylation sites have been suggested as well (Routray et al., 2013), with both LjCCaMK T265 and MtCCaMK T271 being conserved in the kinase domain (Shimoda et al., 2012; Routray et al., 2013). Although the CaMB domain phosphorylation sites S337 and S344, in LjCCaMK and MtCCaMK, respectively, have both been reported to negatively regulate the function of these CCaMKs, these sites are not found at equivalent but consecutive positions in their respective proteins sequences. In addition, these sites have been found to be highly conserved in rhizobial and mycorrhizal plant angiosperm CCaMKs for each case (Liao et al., 2012; Routray et al., 2013). It has been indicated that the decreased kinase activities, that follow the autophosphorylation of these two autophosphorylation sites in the CaMB domain, result in different physiological responses. Further, the authors noted the complexity and delicacy of the regulatory mechanisms involved in the fine-tuning actions of CCaMKs during bacterial and fungal symbioses. However, the conserved autophosphorylation site of the CCaMK kinase domain (T265 and T271 in LjCCaMK and MtCCaMK, respectively) has been considered as critical regulator of the function of CCaMK (Routray et al., 2013).

CCaMK is localized in the nucleus (Smit et al., 2005) and lies downstream of calcium spiking (Miwa et al., 2006) which is induced by the LysM-receptor-like kinases (LysMRLKs) of symbiosis (Sym) pathway (Oldroyd and Downie, 2004). Once activated, CCaMK phosphorylates its downstream substrate CYCLOPS or IPD3 in *M. truncatula*, which is a coiled-coil protein essential for microbial infection in various legumes (Capoen and Oldroyd, 2008; Yano et al., 2008; Singh and Parniske, 2012). Besides function in rhizobial and AM symbiosis, CCaMKs have been shown to be involved in abscisic acid (ABA) signaling during abiotic stress responses (Yang et al., 2011) and ABA-induced antioxidant defense by functioning upstream of ABA-activated MAPK (Shi et al., 2014). However, the role of CCaMKs in plant disease resistance to pathogens has not yet been investigated.

*CCaMK* genes have been identified in various plant species, including nonvascular plant species (Wang et al., 2010) and higher plant species such as monocots (Patil et al., 1995; Asano et al., 2005; Yang et al., 2011), apple (Watillon et al., 1995), tobacco (Liu et al., 1998), *M. truncatula* (Lévy et al., 2004), pea (Lévy et al., 2004), soybean, bean (Zhu et al., 2006), *L. japonicus*, grape (Wang et al., 2010) and *Populus trichocarpa* (Zuo et al., 2013). The CCaMK gene (*DMI3* in *M. truncatula*) was reported to be well-conserved among legume and nonlegume plants that interact with rhizobial bacteria and mycorrhizal fungi, respectively, but no CCaMK ortholog has been found in Arabidopsis which does not establish neither rhizobial nor mycorrhizal symbiosis (Mitra et al., 2004; Zhu et al., 2006). Nevertheless, the degree of CCaMK gene distribution across diverse taxa in the plant kingdom is still unknown.

In this study, we conducted a systemic genome-wide identification of the CCaMKs in genomes of all 44 plant species in Phytozome, revealed their biochemical and gene structural characteristics as well as evolutionary relationship, and analyzed the function of tomato *CCaMK* genes in disease resistance to various pathogens. Our data demonstrate that the structural and biochemical features of CCaMKs are similar among different plant species, and that plant CCaMKs diverge from CDPKs in early ancestor of nonvascular lower land plant species. Additionally, our results reveal that *SlCCaMK* is involved in tomato disease resistance to various pathogens probably via regulating ROS accumulation. This is the first report to demonstrate a role of a *CCaMK* gene in plant disease resistance.

## Materials and methods

### Identification of CCaMKs in plant species

To identify *CCaMK* genes in plant species whose genome sequences are deposited in Phytozome (http://phytozome.jgi.doe.gov/pz/portal.html), a CCaMK from *Lotus japonicus* (LjCCaMK, AM230792, GenBank) was used to BLASTp search against the Phytozome genome databases. All non-redundant sequences with high similarity to LjCCaMK were collected, and subjected to domain analysis using Prosite programs (http://prosite.expasy.org/). A sequence was considered as a CCaMK candidate if it displayed a Ser/Thr kinase domain and three EF-hand motifs as suggested previously (Harmon et al., 2000). Full-length protein sequence of *Fragaria vesca* CCaMK (FvCCaMK) was obtained from NCBI (accession number XP_004300049).

### Phylogenetic analyses of plant CCaMKs

A total of 72 protein sequences were used to construct a phylogenetic tree. They were from a wide variety of plant species belonging to various evolutionary positions and included 51 full-length CCaMKs identified in the present and previous studies, 12 CDPKs and 6 CDPK-related kinases (CRKs) from Arabidopsis as well as three apicomplexan CDPKs; TgCDPK1, PfCDPK3, and CpCDPK1 (Table S1). These protein sequences were aligned using MUSCLE program (Edgar, 2004). The phylogenetic tree was constructed using MEGA 5.0 by maximum likelihood (ML) method following JTT model (Jones et al., 1992; Tamura et al., 2011). One thousand bootstrap replicates were performed to evaluate the support of clusters and nodes. The three apicomplexan CDPKs were used as outgroup for construction of rooted tree (Valmonte et al., 2014).

### Gene structure analyses of plant *CCaMKs*

All plant CCaMK genes with available CDS and genomic sequences corresponding to the open reading frame region were used. The exon/intron structures of *CCaMK* genes were analyzed online using the Gene Structure Display Server (GSDS) with default settings (http://gsds.cbi.pku.edu.cn/) (Guo et al., 2007).

### CCaMK protein sequence comparison and myristoylation prediction

The CCaMK protein sequence alignments of the kinase domain N-terminus and CaMB domain were compared by generating the sequence logos using the Geneious software (v8.1.6) package (http://www.geneious.com/). The N-terminal myristoylation of CCaMK proteins were predicted using an N-terminal myristoylation prediction tool on ExPASy with default settings (http://web.expasy.org/myristoylator/) (Bologna et al., 2004).

### Plant materials for expression analysis

Tomato plants were grown in growth chambers at 28°C with a 16 h/8 h light/dark daily cycle. *Sclerotinia sclerotiorum* was cultured at 25°C on potato dextrose agar (PDA) plates for 2 days. PDA plugs of 5 mm in diameter containing the most active young mycelia were taken from the outside circle of the colonies and were inoculated on the fully developed leaves of the 7~8-week-old tomato plants. 500 μM of OA was prepared and infiltrated into leaves of 7~8-week-old tomato plants. *Pseudomonas syringae* pv. *tomato* (*Pst*) DC3000 and *Xanthomonas oryzae* pv. *oryzae* (*Xoo*) were incubated on King's B medium containing rifampicin (50 μg/ml) and NA medium containing carbenicillin (50 μg/ml) overnight at 28°C, respectively. The bacterial cells were collected by centrifugation after shaking overnight and then diluted into suspensions to a concentration of OD_600_ at 0.002 and 0.5 with 10 mM MgCl_2_ buffer or sterilized ddH_2_O, respectively. The prepared bacterial suspensions (with 10 mM MgCl_2_ buffer or sterilized ddH_2_O as controls) were infiltrated into leaves of tomato plants. Samples were collected for gene expression analysis at two time-points after inoculation or treatment; 0 and 12 h for *S. sclerotiorum*; 0 and 4 h for OA treatment; 0 and 4 h for *Pst*DC3000 as well as 0 and 8 h for *Xoo* inoculation, respectively. At least six plants were used for each treatment. The experiments were repeated at three times.

### Gene expression analyses by RT-qPCR

Real time quantitative RT-PCR (RT-qPCR) analyses and consequent statistical data analyses were conducted as described (Zhao et al., 2013). The following primers were used in RT-qPCR analyses for the *SlCCaMK* gene: E*SlCCaMK*-F (5′-GTAATCAATCAATTAGATCA-3′) and E*SlCCaMK*-R (5′-GTCTTAATTGCAACAACTTC-3′). To normalize the sample loading variance, *SlrRNA* gene served as the internal control with primers *SlrRNA*-F (5′- GCCGGCGACGCATCATTCAAA-3′) and *SlrRNA*-R (5′- CGCGCCTGCTGCCTTCCTT-3′). The expression analyses were conducted three times independently. Data were analyzed using SPSS (verson19.0) by Student's *t*-test (*P* value ≤ 0.05).

### VIGS manipulation procedure and plant disease resistance analysis

VIGS analysis of the *SlCCaMK* gene was conducted as described (Zhao et al., 2013). The VIGS vector pYL156 (pTRV2) containing the SlCCaMK-VIGS fragments were electroporated into *Agrobacterium tumefaciens* strain GV3101. VIGS analyses were conducted with the vacuum-infiltration delivery approach. The agro-infiltrated plants were grown in plant growth chamber at 21°C with a 16 h/8 h light/dark regime. Three weeks later, leaves were sampled to check the gene silencing efficiency and the plants were subjected to disease resistance analyses. Gene silencing efficiency was calculated as the percentage of reduced accumulation of gene transcript in silenced plants over the non-silenced control plants. Accumulation of gene transcript was analyzed by RT-qPCR with the primers V*SlCCaMK*-F (5′-Aggtacc CAAGATGTTGTACTATCCTC-3′) and V*SlCCaMK*-R (5′-Cgaattc TACATATATATGATTAACTA-3′). The silenced and control plants were inoculated with host pathogens *S. sclerotiorum* and *Pst* DC3000 and nonhost pathogen *Xoo* as described above. For necrotrophic pathogen *S. sclerotiorum*, lesion diameter was recorded, while for the bacterial pathogens, the bacterial number inside the inoculated leaf areas was counted as described previously (Li et al., 2012). For each pathogen at least six silenced plants were examined at each time point. The experiments were conducted three times independently. Data were analyzed using SPSS (verson19.0) by Student's *t*-test (*P* value ≤ 0.05).

### Histochemical detection of H_2_O_2_

Plant leaves infiltrated with *Pst* DC3000 bacterial cells or 10 mM MgCl_2_ buffer were sampled at 12 hpi. H_2_O_2_ was detected *in situ* by DAB (3, 3′-diaminobenzidine) staining as described previously (Li et al., 2012).

## Results

### Systemic identification of *CCaMK* genes in plant genomes

**Figure 1:**
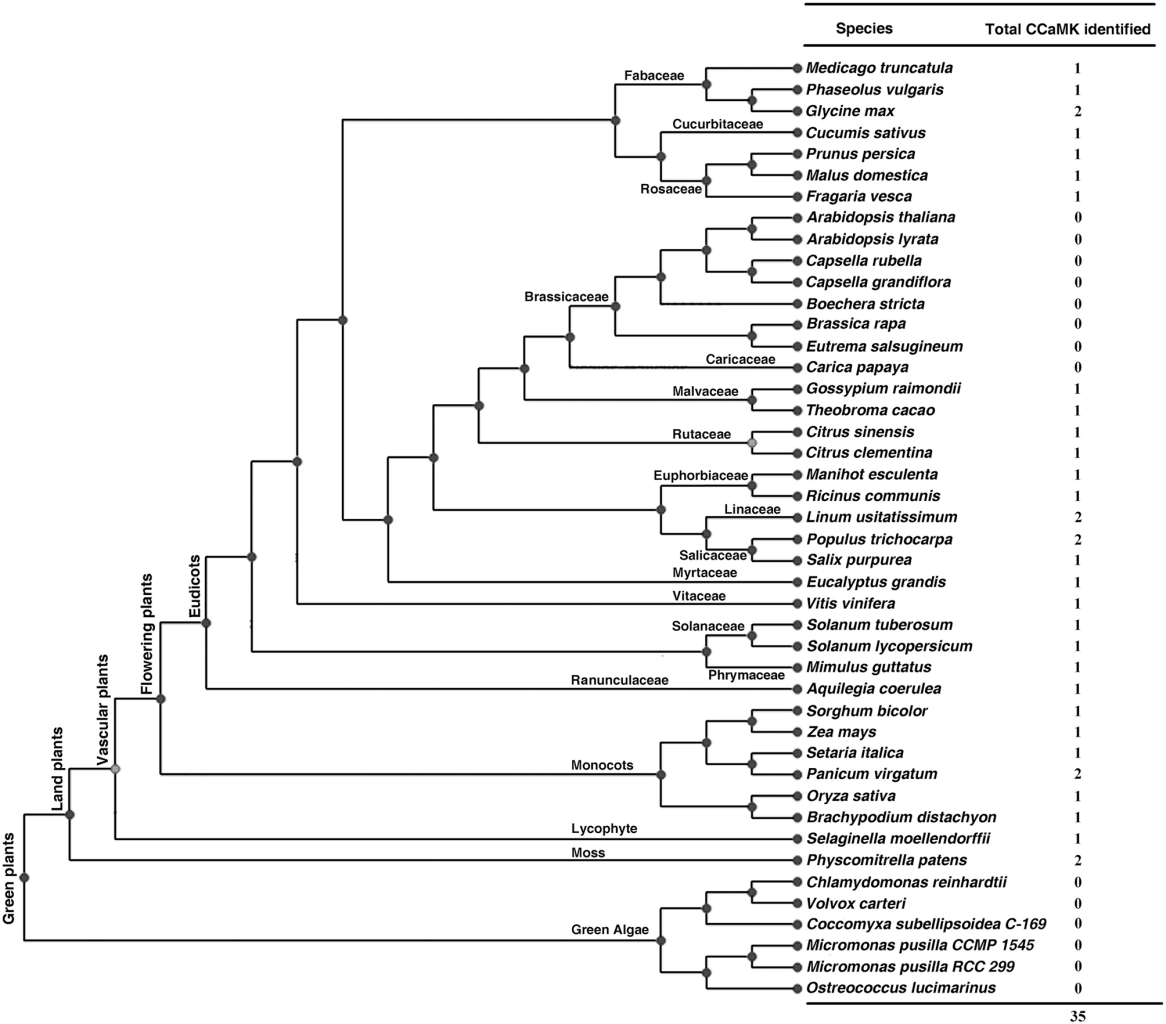
**CCaMKs identified in 44 plant species whose genome sequence data are deposited in Phytozome**. Phylogenetic tree for the plant species is adapted from the Phytozome database (http://phytozome.jgi.doe.gov/pz/portal.html).

To identify *CCaMKs* in plants, a well-known CCaMK from *Lotus japonicus* (LjCCaMK, AM230792 in GenBank) was used for BLASTp search against genome of all 44 green plant species deposited in the Phytozome database (version 10.3, http://phytozome.jgi.doe.gov/pz/portal.html) (Figure 1). Consequently, 38 non-redundant sequences with high similarity (*e* value < e-110) to LjCCaMK were retrieved and their domain composition was analyzed by Prosite programs (http://prosite.expasy.org/). Those containing a Ser/Thr kinase domain and three EF-hand motifs were recognized as CCaMKs as suggested previously (Harmon et al., 2000). Thirty four protein sequences from 29 plant species fulfilled this criterion and thus considered as CCaMKs (Figure 1). The remaining four sequences were found to be different from canonical CCaMKs. These included two from *Carica papaya* (evm.TU.supercontig_54.31 and evm.TU.supercontig_1289.1), one from *Fragaria vesca* (gene29272-v1.0-hybrid) and one from *Zea mays* (GRMZM2G177475). The two *C. papaya* and one *F. vesca* sequences were significantly shorter than canonical CCaMKs, and thus thought truncated. However, based on the alignment with full length CCaMKs from other species, the two *C. papaya* sequences seemed to be two parts of a complete CCaMK (Supplementary Figure 1, discussion part). In addition, a full-length *F. vesca* CCaMK was obtained from NCBI (accession number XP_004300049). The sequence from *Z. mays* possessed similar size as canonical CCaMKs but only carried an EF-hand domain and thus might be not a CCaMK protein. Among the total 35 full-length CCaMK proteins from 29 flowering plant species, 18 of which were reported to contain CCaMK genes for the first time in this study (Figure 1, Table 1). Remarkably, among the 44 Viridiplantae species examined in this study, 13 species were found to contain no CCaMK protein sequence. These included all checked seven cruciferous species (*Arabidopsis lyrata, A. thaliana, Boechera stricta, Brassica rapa FPsc, Capsella grandiflora, C. rubella*, and *Eutrema salsugineum*) and six algal species (*Volvox carteri, Coccomyxa subellipsoidea C-169, Micromonas pusilla CCMP1545, Micromonas sp. RCC299, Chlamydomonas reinhardtii, Ostreococcus lucimarinus*, and *O. tauri*). Moreover, contrary to the previous suggestion that CCaMKs (DMI3) may occur in all land plants in single copy (Wang et al., 2010), genomes of some plant species such as *P. patens, Panicum virgatum, P. trichocarpa, Linum usitatissimum*, and *Glycine max*, were found to contain two CCaMK gene copies (Figure 1), apparently resulting from independent genome duplications.

**Table 1:** **The 18 plant species in which *CCaMK* genes were newly identified in this study**.

**Species**	**Protein**	**Accession number (Phytozome)**	**Number of EF-hand motifs**	**Protein size (aa)**	**Mol Wt (kDa)**	**pI**	**Intron number**	**N-terminus**	**N-myristoylation^a^**
**MONOCOTS**									
*Panicum virgatum*	PvCCaMK1	Pavir.J01749.1	3	513	56.93	5.66	6	MSKTESRKLS	NO
	PvCCaMK2	Pavir.Ab00605.1	3	499	55.10	5.57	5	MSKTESRKLS	NO
*Setaria italica*	SiCCaMK	Si021787m	3	513	57.02	5.57	6	MSKTESRKLS	NO
**DICOTS**									
*Aquilegia coerulea*	AcCCaMK	Aquca_093_00002	3	522	57.73	5.49	6	MGHETRRLSD	NO
*Mimulus guttatus*	MgCCaMK	Migut.N01872	3	512	57.37	5.63	6	MEQGTRKSIT	NO
*Solanum lycopersicum*	SlCCaMK	Solyc01g096820.2.1	3	516	57.90	5.38	7	MGGLDVIRTS	NO
*Solanum tuberosum*	StCCaMK	PGSC0003DMT400070801	3	515	57.67	5.16	7	MGEKDVIRTS	NO
*Eucalyptus grandis*	EgCCaMK	Eucgr.G02633.1	3	516	57.76	5.20	6	MDQETRRLSD	NO
*Salix purpurea*	SpCCaMK	SapurV1A.0036s0860.1	3	550	62.01	5.46	5	MGQETRRLSD	NO
*Linum usitatissimum*	LuCCaMK1	Lus10033400	3	520	58.13	6.30	6	MGKETKRLVD	NO
	LuCCaMK2	Lus10034860	3	518	58.16	6.30	6	MGKETKRLVD	NO
*Manihot esculenta*	MeCCaMK	cassava4.1_026542m	3	507	57.04	5.63	6	MGQKTRKLSD	NO
*Ricinus communis*	RcCCaMK	30226.m002047	3	508	56.88	5.49	6	MGQKSKKLSD	NO
*Gossypium raimondii*	GrCCaMK	Gorai.N006600.1	3	520	57.78	5.63	6	MGQDKAKLVE	NO
*Theobroma cacao*	TcCCaMK	Thecc1EG044590t1	3	524	58.27	5.31	6	MGQEKGKLCD	YES (0.148523)
*Citrus sinensis*	CsCCaMK	orange1.1g009980m	3	521	58.32	5.05	5	MGQETRKLTD	NO
*Citrus clementina*	CcCCaMK	Ciclev10000859m	3	522	58.34	5.05	5	MGQETRKLTD	NO
*Cucumis sativus*	CsaCCaMK	Cucsa.364320.1	3	517	57.80	5.90	6	MIQQARKLSE	NO
*Fragaria vesca*^b^	FvCCaMK	XP_004300049	3	523	58.21	5.55	6	MGQETRRLAD	NO
*Prunus persica*	PpeCCaMK	ppa004207m	3	523	58.08	5.62	6	MGQETRRLAD	NO

a *The score for prediction of existence of N-myristoylation motif: 0 < Score < 0.4 (Low Confidence), 0.4 < Score < 0.85 (Medium Confidence), 0.85 < Score < 1 (High Confidence)*.

b *The Fragaria vesca CCaMK full-length protein sequence was obtained from NCBI*.

To identify more CCaMKs especially in more lower plant species in addition to those from the 44 green plant species whose genome sequences were deposited in Phytozome, we analyzed the domain composition of the candidate CCaMK protein sequences listed at Table 1 by Wang et al. (2010). Among the 41 sequences listed, 22 are full length sequences that contained both a kinase domain and an EF-hand CaMB domain and thus were recognized as full-length CCaMKs. These CCaMKs were from diversified lower nonvascular liverworts, mosses and hornworts, vascular non-flowering plant species and higher flowering plant species (Table S1). This result further demonstrates that CCaMKs are widely distributed in various lower plant species.

### Phylogeny of plant CCaMKs

**Figure 2:**
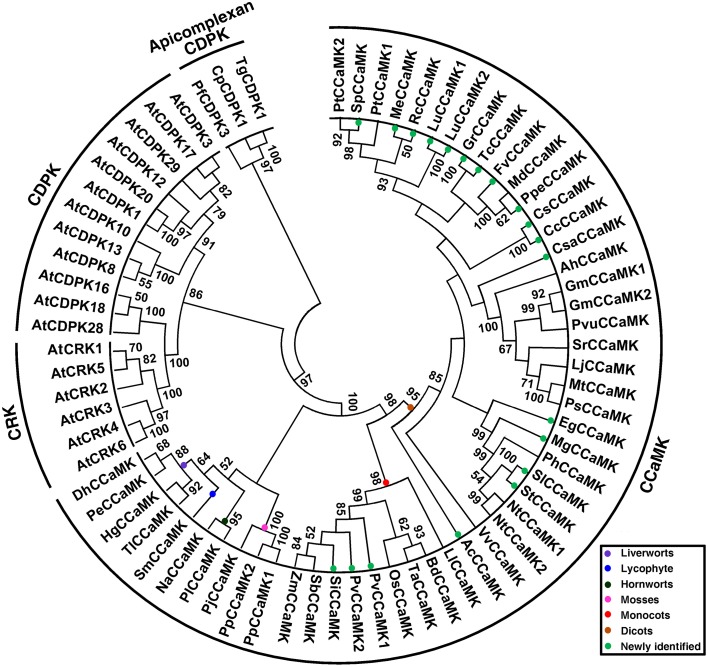
**Phylogenetic tree of plant CCaMK proteins**. The tree was created based on alignment of full-length proteins using maximum likelihood (ML) method with bootstrap of 1000 in MEGA 5.0. Fifty-one CCaMKs identified from a wide variety of plant species at diversified evolutionary positions together with 21 CDPKs and CRKs (Table S1) were subjected to tree construction. Three apicomplexan CDPKs, TgCDPK1, PfCDPK3, and CpCDPK1, were included as outgroup. Different clades of CCaMKs from various taxonomic groups are indicated in different colors. The 18 species for which CCaMKs genes are newly identified in this study are marked with solid green circles.

To understand the phylogeny of plant CCaMKs, all newly identified CCaMK sequence candidates and those of previous reports, and thus from a wide variety of plant species at different evolutionary positions, were subjected to phylogenetic analysis along with Arabidopsis CDPK and CRK representatives (Table S1). In the rooted maximum likelihood (ML) phylogenetic tree using apicomplexan CDPKs as outgroup, all CCaMK sequences clustered together with obvious separation from CDPKs and CRKs (Figure 2). The clustering of plant CCaMKs reflected the evolutionary relationship among the plant species. According to the ML tree, the 51 plant CCaMKs were classified into three obviously distinct groups, i.e., lower land plant species group, monocot group and dicot group, with strong bootstrap support (Figure 2). The group of the lower plant species CCaMKs comprised sequences from hornwort, moss, liverwort and lycophyte, the monocot group consisted of Liliaceae and Poaceae *CCaMK*s, while within the dicot group clustered the CCaMKs from *Aquilegia coerulea* and all the dicot species (Figure 2). This result indicates that the *CCaMK* lineage may have diverged from a CDPK gene in the earliest multicellular ancestor of all land plant species, considering that CCaMKs widely exist in nonvascular plant species but not in unicellular algal species.

### Conservation of domain composition and key regulatory residues of plant CCaMKs

CCaMKs should contain at least a protein kinase domain, autoinhibitory/CaMB domain and a visinin-like EF-hand calcium-binding domain. We checked domains of 51 CCaMK proteins used in the phylogenetic tree construction. Result showed that, as expected, all these CCaMKs harbored a Ser/Thr kinase domain and an EF-hand calcium-binding domain with three Ca^2+^ binding sites, except one sequence from *P. trichocarpa* (Potri.010G247400.1) that has lost one EF-hand motif (Table 1, Table S1). This indicated that the domain composition of CCaMKs is highly conserved among all land plant species. Moreover, unlike most of CDPKs and CRKs, which are associated with membrane through myristoylation, bioinformatics prediction showed that all but one of the newly identified CCaMKs did not bear an N-terminal myristoylation motif with exception to a *Theobroma cacao* CCaMK. However, the N-terminal myristoylation motif in this sequence was predicted with very low confidence (Table 1). This suggested that plant CCaMKs and CDPKs might have distinct cellular localization. Furthermore, *in silico* prediction revealed high conservation of biochemical characteristics among CCaMK proteins. The CCaMK proteins newly identified in this study comprised 499~550 amino acids with molecular weight of 55.10~62.01 kDa and a predicted pI value of 5.05~6.30 (Table 1), demonstrating that all these CCaMKs are acidic proteins.

**Figure 3:**
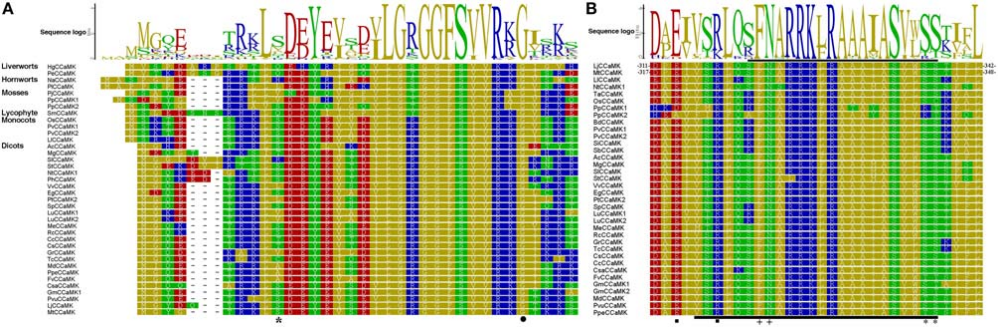
**Protein sequence logos for the kinase domain N-terminal and autoinhibitory/CaM-binding domain of CCaMK proteins**. **(A)** Shows the logo for N-terminal of the kinase domain of sequences from various plant species. **(B)** Indicates the autoinhibitory domain overlapping with the CaM-binding domain. These two domains are shown by the black bar below and above the alignment, respectively. The two of autoinhibition stabilizing residues are shown by the solid black boxes, CaM-binding site is indicated by “plus” sign, CCaMK autophosphorylation sites are indicated by “asterisk,” while the position of G30 in LjCCaMK is indicated by the solid black circle.

Additionally, we compared N-terminal of the kinase domain containing glycine-rich region in CCaMKs from different plant species. The result demonstrated that the residue G30 in LjCCaMK, required for CCaMK kinase activity (Shimoda et al., 2012), is conserved in all plant CCaMKs (Figure 3A), except for PtCCaMK1 sequence in which the corresponding region is deleted (Supplementary Figure 1). Notably, the phosphorylation site S9 in MtCCaMK, located near this region (Routray et al., 2013), seems to be generally conserved in all monocots and fabaceae with an overall conservation of 56% of the total 51 CCaMKs analyzed. While *P. patens* is the only lower plant species with CCaMK to possess serine residue at similar position, it is substituted by threonine (which can also be phosphorylated) in three sequences (T9 in the *C. clementina* and *C. sinensis* CCaMKs and T10 in the *M. guttatus* CCaMK), and arginine in the tomato sequence (R10) which is an exception to the other solanaceae CCaMKs (Figure 3A). More importantly, further comparison of the remaining part of the kinase domain revealed that major and preferable autophosphorylation site (T265 LjCCaMK and T271 in and MtCCaMK), which is the crucial regulator of the CCaMK function (Routray et al., 2013), is conserved besides its substitution for serine in five CCaMKs from non-vascular plants (mosses and two liverworts *Dumortiera hirsute* and *Pellia epiphylla*), and the two from the higher plant dicot *Linum usitatissimum* (Supplementary Figure 1).

Comparison of autoinhibitory/CaMB domain of CCaMKs identified in this work and previously reported representatives demonstrated high level of conservation. Overall, residues involved in CaM binding (F321N322 in LjCCaMK) are 100% conserved across all CCaMKs (Figure 3B). In addition, the key autophosphorylation sites, located at the end of CaMB domain, required for negative autoregulation of either LjCCaMK (S337) or MtCCaMK (S344) are highly conserved, or substituted by an equally phosphorylable residue threonine, in all CCaMKs with only a few exceptions such as in moss, spike moss (*Selaginella moellendorffii*) and rice. It is noteworthy to mention that S337 of LjCCaMK and S344 of MtCCaMK are not equivalent. Instead, they are located at consecutive positions in each CCaMK protein (Figure 3B). In moss sequences, there is an ariginine residue at equivalent position of S337 in LjCCaM (R324 in PpCCaMK1 and R330 in PpCCaMK2), while spike moss and rice sequences contain asparagine and cystein, respectively, at position corresponding to S344 in MtCCaMK (Figure 3B). The autoinhibitory domain contains one of the amino acid residues (R317 in LjCCaMK) implicated in a hydrogen-bond network which maintains the inhibition in the absence of Ca^2+^ (Shimoda et al., 2012). This residue is conserved in all lower plants and dicots, while it is substituted by lysine in all monocots. Another residue of this network (E313 in LjCCaMK) is located near the autoinhibitory domain and is conserved as well-except in the two PpCCaMKs, where it was shifted one position before in PpCCaMK2 (E305) and replaced by aspartate in PpCCaMK1 (D300) (Figure 3B). The remaining two residues in the hydrogen-bond network (S237 and K264 in LjCCaMK) are located in the kinase domain, with S237 being conserved or replaced by cystein in lower plants while the K264 corresponding residues show basically lineage dependent variations (Supplementary Figure 1). All together, these results suggest that the differential responses among plant CCaMKs is likely to result from distinctively phosphorylated residues.

### Exon/intron structure of plant *CCaMK* genes

**Figure 4:**
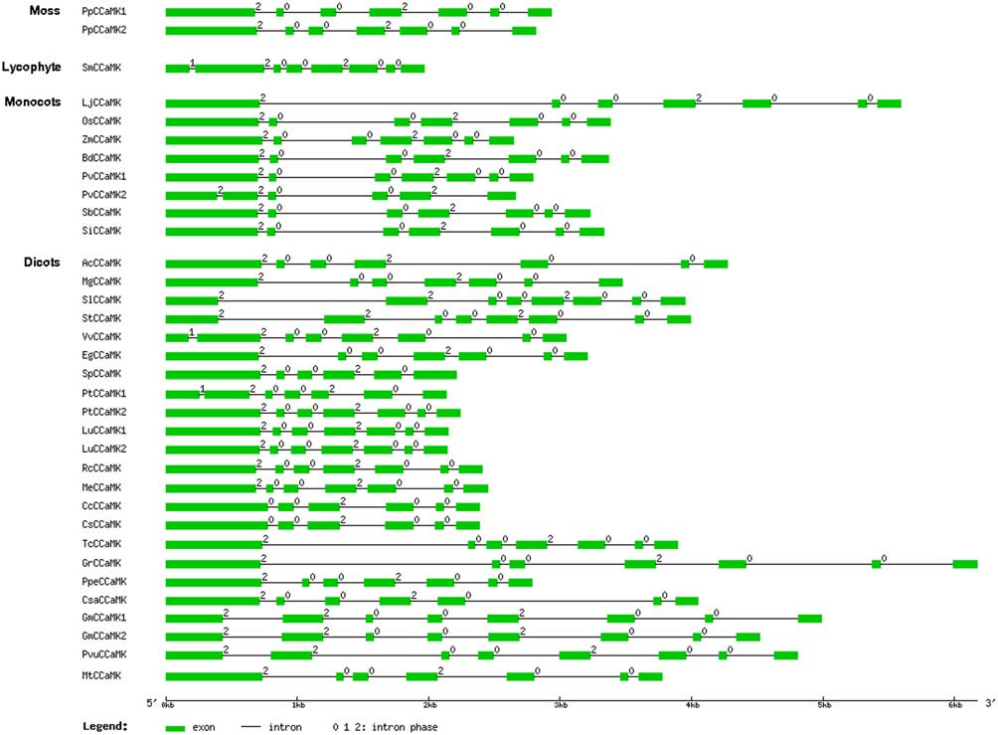
**Schematic diagram indicating the exon/intron structure of the *CCaMK* genes**. The locus number of the 34 *CCaMK* genes was listed in Table S1. Exons and introns are indicated as green boxes and black lines, respectively. The intron phase number 0, 1, and 2 are labeled at the beginning of each intron. The diagram is drawn to scale.

To further understand the relationship among the *CCaMK* genes from different plant species, we examined the exon/intron structure of all the 34 *CCaMK* genes whose full-length protein and genomic sequences were both available in the Phytozome database. They were from various plant species at diversified evolutionary positions (Figure 1, Table S1). Results showed that the intron number and phase pattern of the *CCaMK* genes were similar among different plant species (Table 1, Figure 4). Most (23/34) *CCaMK* genes, including the *PpCCaMK1* and *PpCCaMK2* from moss (*P. patens*), carried 6 introns with a phase pattern of 200200 except the *PtCCaMK1* gene whose intron phase pattern was 120020. The remaining 11 *CCaMK* genes contained 5 or 7 introns. Among these, five 7-intron genes from solanaceous species tomato and potato and leguminous species soybean and bean, carried an intron phase profile of 2200200, while the remaining two 7-intron genes from a lycophyte (*Selaginella moellendorffii*) and a dicot (*Vitis vinifera*) possessed an intron phase profile of 1200200. The four 5-intron genes were from four species with various intron phase patterns. They include *PvCCaMK2* from *Panicum virgatum*, with an intron phase pattern of 22002, *SpCCaMK* from *Salix purpurea* with an intron phase pattern of 20020, and two citrus genes *CsCCaMK* and Cc*CCaMK* with an intron phase pattern of 00200 (Table 1, Figure 4). Notably, the difference in the intron number of *CCaMK* genes mainly resulted from the first exons. Collectively, the exon/intron structures of the plant *CCaMK* genes are generally conserved and fits the clustering of the *CCaMK* phylogenetic tree.

### Expression of *SlCCaMK* was highly responsive to diverse stimuli

**Figure 5:**
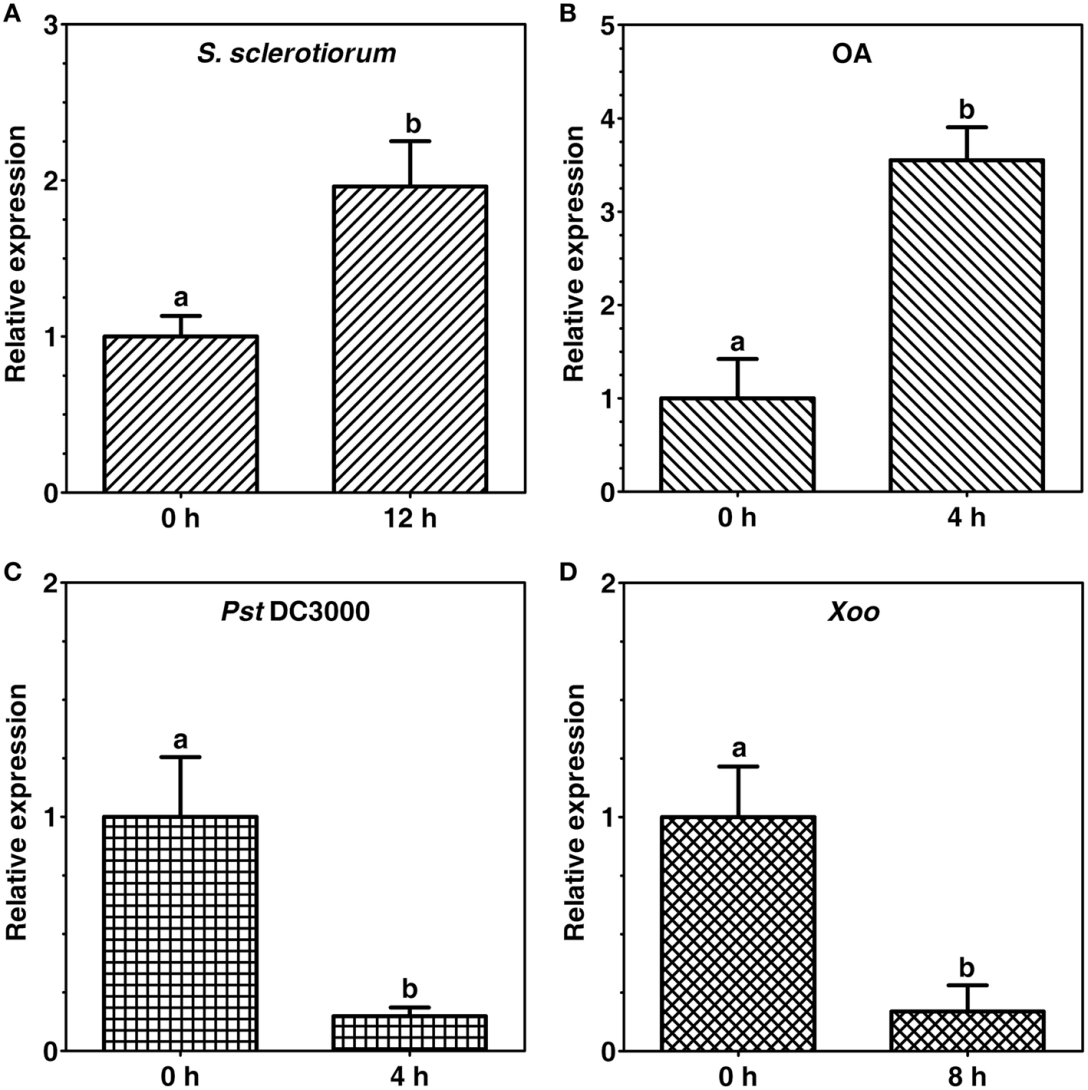
**Expression patterns of the *SlCCaMK* gene in response to pathogen inoculation and pathogenecity factor treatment**. Gene expression was analyzed at 12 h after *S. sclerotiorum* inoculation **(A)**, 4 h after OA treatment **(B)**, 4 h after *Pst* DC3000 infiltration **(C)**, and 8 h after *Xoo* infiltration **(D)**. The small letters indicate the signicant difference of the *SlCCaMK* gene expression under each stimulus (*P* ≤ 0.05, by Student's *t*-test).

Expression of tomato *CCaMK* (*SlCCaMK*) in response to host pathogens [*Sclerotinia sclerotiorum, Pseudomonas syringae* pv. *tomato* (*Pst*) DC3000] and a non-host pathogen (*Xanthomonas oryzae* pv. *oryzae, Xoo*) as well as a pathogenicity factor of the pathogen *S. sclerotiorum*, oxalic acid (OA), in tomato were investigated. Expression of the *SlCCaMK* was up-regulated by about 2 folds at 12 h post inoculation (hpi) of *S. sclerotiorum* (Figure 5A) and by 3.6 folds at 4 h after infiltration treatment with OA (500 μM), which is produced during plant infection with *S. sclerotiorum* (Figure 5B). In contrast, expression of the *SlCCaMK* was strongly suppressed by about 85% both at 4 hpi with the bacterial host pathogen *Pst* DC3000 (Figure 5C) as well as at 8 hpi with a non-host bacterial pathogen *Xoo* (Figure 5D). These data indicate that expression of the *SlCCaMK* gene is widely and diversely responsive to a variety of pathogen and pathogen-related stimuli and imply that this gene may be involved in tomato resistance to these pathogens.

### Knock-down of *SlCCaMK* reduced the resistance to *S. scelrotiorum* and *Pst* DC3000 in tomato

**Figure 6:**
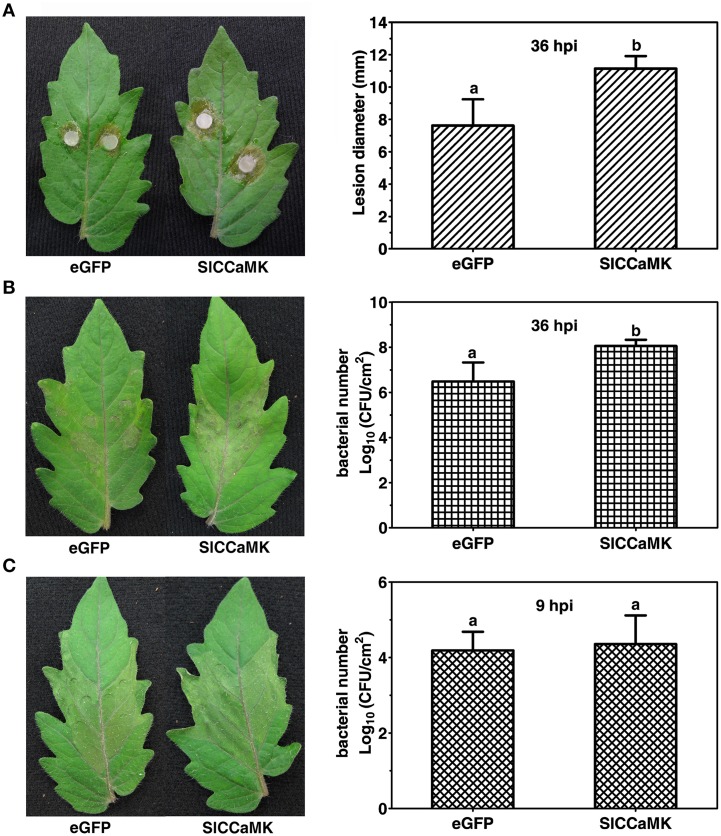
**Knock-down of the *SlCCaMK* gene by VIGS decreased the tomato disease resistance**. **(A)** The necrosis symptoms caused by *S. sclerotiorum* inoculation and statistical analysis of lesion diameter at 36 hpi. **(B)** The necrosis symptoms caused by *Pst* DC3000 infiltration and bacterial number in the infiltrated areas at 36 hpi. **(C)** The HR symptoms caused by *Xoo* infiltration and bacterial number in the infiltrated areas at 9 hpi. Significant differences of bacterial number and lesion diameter are indicated as different lowercase letters (*P* ≤ 0.05, by Student's *t*-test).

To further understand the function of the *SlCCaMK* gene in plant disease resistance, VIGS analyses were performed for the *SlCCaMK* gene. A vector containing a fragment of eGFP was used as the control in agro-infiltrated plants (Zhao et al., 2013). Three weeks post agro-infiltration, the VIGS-treated (VT) tomato plants were inoculated with the host pathogens *S. sclerotiorum* and *Pst* DC3000 and the nonhost pathogen *Xoo*, and thereafter the resistance was evaluated. When inoculated with *S. sclerotiorum*, the *SlCCaMK*-VT plants displayed more severe disease symptom than the eGFP-control plants. The lesion diameter of these plants was 11.1 mm at 36 hpi, which was significantly larger than that of eGFP-control plants (7.6 mm) (Figure 6A). This result indicated that *SlCCaMK* may play a positive role in basal resistance to *S. sclerotiorum*. In case of inoculation with *Pst* DC3000, the *SlCCaMK*-VT plants exhibited stronger necrosis disease symptom at 36 hpi than the eGFP-control plants. Meanwhile, the bacterial number on these plants was about 1.5 orders of magnitude higher than the control plants (Figure 6B). This result showed that *SlCCaMK* may be positively involved in basal resistance to *Pst* DC3000. When inoculated with *Xoo*, the *SlCCaMK*-VT plants did not show obvious difference in either HR symptom or bacterial number in the *Xoo*-infiltrated areas in comparison with the control plants (Figure 6C). This implied that VIGS treatment of the *SlCCaMK* gene may have no influence on tomato resistance to *Xoo*.

**Figure 7:**
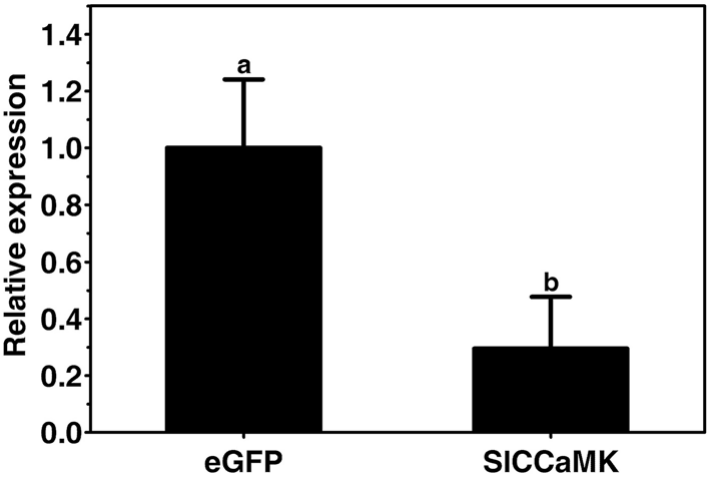
**Evaluation of gene silencing efficiency**. Expression levels of the *SlCCaMK* gene in tomato plants were examined by RT-qPCR. Significant difference between expression values of the *SlCCaMK* gene in silencing-treated plants and those of the eGFP control plants is indicated as a lowercase letter (*P* ≤ 0.05, by Student's *t*-test).

To ensure the silencing efficiency of the *SlCCaMK* gene, the expression of the *SlCCaMK* gene in the VIGS-treated and non-silenced eGFP control plants was compared. Results of RT-qPCR analysis showed that transcript of the *SlCCaMK* gene in the VIGS-treated plants accumulated only 29.5% of that of control plants (Figure 7), indicating this gene was effectively knocked down, and the observed altering in disease resistance is attributed to the *SlCCaMK* gene.

Collectively, these results revealed that *SlCCaMK* is required for basal resistance to both *S. sclerotiorum* and *Pst* DC3000, and indicate that CCaMKs might play important roles in plant disease resistance to diverse pathogens.

### Knock-down of *SlCCaMK* decreased ROS accumulation in response to *Pst* DC3000 inoculation in tomato

**Figure 8:**
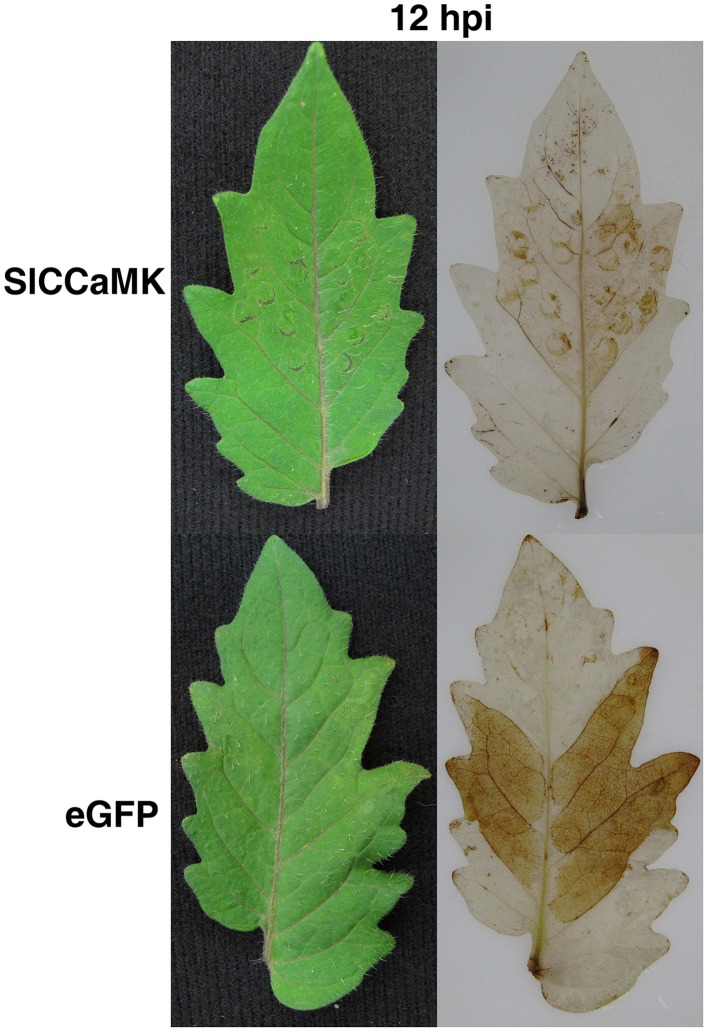
**Knock-down of the *SlCCaMK* gene by VIGS reduced H_2_O_2_ accumulation**. Tomato leaves of the *SlCCaMK*-silenced and the eGFP control plants were collected at 12 h post infiltration with *Pst* DC3000 or with sterilized water as a control, and stained with DAB. Leaves both before (Left panels) and after (Right panels) staining were shown.

To understand how the *SlCCaMK* regulates plant disease resistance, effect of gene knock-down on ROS accumulation was examined through VIGS analyses. At 12 h post inoculation with *Pst* DC3000, the infiltrated leaf areas of the non-silenced eGFP control plants were stained deep brown, indicating that these leaves accumulated high level of H_2_O_2_. However, the infiltrated leaf areas of the *SlCCaMK*-knock-down plants were only very weakly stained, demonstrating that these leaves accumulated very low level of H_2_O_2_ (Figure 8). This result revealed that *SlCCaMK* play a positive role in regulating H_2_O_2_ accumulation.

## Discussion

CCaMKs are plant-specific functioning in establishment of rhizobial and arbuscular mycorrhizal (AM) symbioses. They have been identified in a number of both legume and non-legume plant species. Of all the identified CCaMK sequences, *L. japonicus* CCaMK and *M. truncatula* CCaMK (DMI3) are the most studied and have been shown to be decoder and transducer of the calcium spiking signals resulting from rhizobial bacteria or AM fungal infections (Wang et al., 2010; Shimoda et al., 2012). However, Arabidopsis is the only asymbiotic plant species with complete sequenced genome that has been found to lack the CCaMK gene (Zhu et al., 2006; Wang et al., 2010). Nevertheless, whether the absence of this gene in Arabidopsis is exceptional is unclear, and the distribution of CCaMKs in green plants, especially flowering plants, and their phylogeny are still not completely understood. Moreover, the function of CCaMKs in plant disease resistance has not been studied yet. In this study, we examined CCaMKs from 44 complete sequenced Viridplantae genomes in Phytozome by analyzing their phylogenetic relationship, domain composition and chemical characteristics, as well as gene exon/intron structure. We revealed that, on the contrary to their close relatives CDPKs and CRKs, CCaMKs are less abundant in plant genomes, as previously suspected that CCaMKs (DMI3) may exist as single-copy genes in the genomes of most, if not all, land plants (Wang et al., 2010). More importantly, no CCaMK exists in unicellular algal species and higher flowering cruciferous species. However, the domain composition, chemical characteristics and gene exon/intron structure of all the identified CCaMKs are similar, demonstrating that the CCaMK gene is conserved in various plant species. Furthermore, we report for the first time the role of a *CCaMK* (*SlCCaMK*) in plant disease resistance and ROS accumulation.

### Distribution and phylogeny of plant CCaMKs

CCaMK belongs to calcium/CaM dependent protein kinase superfamily. It is closely related to CDPKs, with differences being the number of EF-hand motifs (four in CDPKs and three in CCaMKs) and the presence of overlapping autoinhibitory and CaMB domains in CCaMKs (Harmon et al., 2000). Nonetheless, we find that the distribution of CCaMK and CDPK in plants differs strikingly. CDPKs exist in all green plants (including unicellular algae, lower nonvascular and higher vascular plant species) and even protists (Hamel et al., 2014; Valmonte et al., 2014). However, CCaMK protein sequence is not present in at least 13 green plant species according to our data. These plant species included all checked seven cruciferous and six algal species (listed in Results part). Regarding CCaMK in *Carica papaya*, two truncated sequences were retrieved from our BLASTp searches. One of them (evm.TU.supercontig_1289.1) contains a truncated kinase domain while the other (evm.TU.supercontig_54.31) showed incomplete kinase domain and three complete EF-hands. Additionally, when aligned with full-length CCaMKs from other species, these two sequences aligned perfectly with respective regions of complete CCaMK protein sequences. Notably, the sequence which aligned with CCaMK C-terminal region (Phytozome ID evm.model.supercontig_54.31) was found to possess autoinhibitory/CaMB domain as well (Supplementary Figure 1), suggesting that *C. papaya* may have a functional CCaMK gene and that the two truncated sequences are likely to result from poor annotation. Collectively, it is now clear that CCaMKs only exist in land plants but is completely lost in all cruciferous species. This is consistent with the previous reports that CCaMKs are associated with plant species known to establish rhizobial and AM mycorrhizal symbioses (Wang et al., 2010; Shimoda et al., 2012); the conclusion initially drawn mainly based on Arabidopsis, as it was the only cruciferous species with complete genome searched for the existence of the gene.

Regarding the phylogeny of CCaMKs, since CCaMK does not exist in unicellular green algal species but widely present in multicellular lower nonvascular land plant species, while CDPKs are generally distributed in algal species, it seems that CCaMKs emerged in nonvascular land plant species evolving from a CDPK gene of the last ancestor of all land plant species. However, unlike CDPKs which have largely expanded through duplications and rearrangements, most CCaMKs exist as single-copy genes, with exceptions to those four aforementioned species. Since gene duplication may result in functional diversification (Wang et al., 2010), the duplicated CCaMK genes in these plant species are likely to have acquired novel functions. Moreover, comparison of CCaMK protein alignments indicated significant differences in protein sequences encoded by duplicated genes (deletion in general), with exception to those from *L. usitatissimum* and *G. max* (Supplementary Figure 1). These differences are also reflected in gene structures with obvious intron gains and exon losses (Figure 4). While *L. usitatissimum* and *G. max* duplicated CCaMK genes show no apparent differences in their structures, both PvCCaMK2 and PtCCaMK1 gained an intron within the corresponding exon 1 in CCaMKs. There was loss of exons 5 and 6 in PvCCaM, part of exon 4 and the entire exon 6 in PtCCaMK1, whereas PpCCaMK2 only lost a part of exon 4. Contrary to the other CCaMK duplicate genes, which are likely to be functional, the likelihood function for PtCCaMK1 is uncertain given that it has lost parts encoding the important glycine-rich of the kinase domain (including G30) and the second EF-hand motif (Supplementary Figure 1). Besides the duplicate genes, recent intron gains in the first exon appear to have occurred in *S. moellendorffii, V. vinifera*, and the lineages leading to the solanaceae and phaseoleae (*G. max* and *P. vulgaris*). On the other hand, the first exon in the CCaMK genes from Rutaceae species is obviously the fusion product of exons 1 and 2 of the original gene (Figure 4). While these differences in CCaMK gene structures are not surprising due to unequal crossing-over within the same species or mutation across species during evolution, their relevancy to gene function is not yet known.

Studies have proved that plant CCaMKs were under purifying selection for maintenance of their ancestral functions in all mycorrhizal plant lineages (Wang et al., 2010). Consequently, all CCaMKs exhibit similar physico-biochemical properties and are generally highly conserved all along the whole protein sequences, except some few N-terminal regions. On the other hand, studies demonstrated that there is specificity for both rhizobial and mycorrhizal symbioses which results in differential transcriptional gene expression patterns (Singh and Parniske, 2012). However, how CCaMKs interprets the origin of Ca^2+^ spiking signal (whether from rhizobial bacteria or AM fungi) is still poorly understood. In addition, our result on comparison of autoinhibitory/CaMB domain of the CCaMK protein sequences from various plant species, the major autophosphorylation sites and autoinhibitory stabilizing residues (S237, K264, E313 and R317 in addition to T265 in LjCCaMK) indicated no significant differences across all plant CCaMKs. Also, besides T265/T271, the two additional autophosphorylation sites identified in the CaM-binding domain (S337 in LjCCaMK and S344 in MtCCaMK) are both present in each CCaMK, exceptions being those mentioned above (Figure 3B, Supplementary Figure 1). Both S337 and S344 have been shown to negatively regulate LjCCaMK and MtCCaMK, respectively, and have epistatic effect on the main autophosphorylation site of the kinase domain (T265 or T271). The lack of S344 MtCCaMK equivalent in OsCCaMK has been partly suspected to be the major reason for the deregulated kinase activity and spontaneous nodulation phenotype observed in OsCCaMK-complemented legume ccamk mutants (Routray et al., 2013). However, considering the conservation of both two autophosphorylation sites in most of CCaMKs, their individual roles in activity of these proteins remain unclear. Conversely, the autophosphorylation site S9 of MtCCaMK is not conserved in all sequences. Also, there are other serine or threonine residues with considerable degree of conservation in the vicinity of this position (Figure 3A), suggestive for differences in regulation of CCaMK activities through phosphprylation at various distinct phosphorylation sites. It is interesting to note that the protein region containing the position corresponding to S9 in MtCCaMK is encoded by the first exon which also showed some differences in the otherwise conserved CCaMK gene structures (Figure 4). However, it remains to determine the significance of this exon in defining the downstream responses to specific upstream inducers.

Taken together, our results are supportive for CCaMK specific function in mediating microbial-plant symbioses, but the mechanisms underlying the infection-specific activation of its downstream responses remains to be characterized.

### Functions of *SlCCaMK* in plant disease resistance

Plant CCaMKs play a pivotal role in symbiosis establishment with rhizobia and AM fungi (Singh and Parniske, 2012). It is known that some mechanisms of symbiosis and pathogenesis exist in both bacteria and fungi for successful host colonization (Hentschel et al., 2000; García-Garrido and Ocampo, 2002). Nevertheless, whether CCaMKs play a role in plant disease resistance remains unknown. To gain more information about CCaMK gene function in plant disease resistance, we examined expression patterns of tomato *CCaMK* (*SlCCaMK*) gene in response to various plant pathogens. Furthermore, we performed VIGS functional analyses to reveal its role in tomato resistance to three pathogens including *S. sclerotiorum, Pst* DC3000 and *Xoo*, representing three different types of resistance, host basal resistance to necrotrophic fungal pathogen, host basal resistance to biotrophic bacterial pathogen, and nonhost resistance to bacterial pathogen, respectively. The results showed that the expression of *SlCCaMK* is stimulus dependent. It is induced by *S. sclerotiorum* and OA, but strongly suppressed by *Pst* DC3000 and *Xoo* (Figure 5). Furthermore, VIGS functional analyses reveal that *SlCCaMK* gene is involved in basal resistance to both *S. sclerotiorum* and *Pst* DC3000 (Figure 6). To our knowledge, this is the first report on the role of *CCaMK* in plant disease resistance, and *SlCCaMK* is the first plant *CCaMK* gene that is proved to function in disease resistance.

The function mechanism of *SlCCaMK* to regulate plant resistance to these pathogens remains unclear. However, its involvement in disease resistance seems to be associated with a complex cross-talk and antagonizing actions among defense phytohormones and their regulatory effects on transcription factors. CCaMK interacts and phosphorylates downstream CYCLOPS/IPD3 which subsequently binds the promoter and activates the transcription of the of target genes including NODULE INCEPTION (NIN) (Singh et al., 2014; Gobbato, 2015) and Ethylene Responsive element binding Factor (ERF) Required for Nodulation (ERN) (Middleton et al., 2007; Singh and Parniske, 2012). Interestingly, members of ERF family have been shown to be induced in response to pathogen infection, mechanical wounding, and abiotic stresses as well as specifically by defense signaling molecules including ethylene (ET), jasmonic acid (JA), and/or salicylic acid (SA) (Kloppholz et al., 2011). Ethylene generally works together with JA in the resistance responses to necrotrophic pathogens (Derksen et al., 2013) including *S. sclerotiorum* (Guo and Stotz, 2007; Perchepied et al., 2010). Therefore, the role of SlCCaMK in defense against *S. sclerotiorum* is probably achieved through activation of ERF transcription factors. Consistent with this, overexpression of some ERF transcription factors, such as soybean GmERF3 gene in tobacco, has been found to increase resistance to pathogen attack (Zhang et al., 2009). Additionally, these data show that *Pst* DC3000 pathogenecity on tomato is probably similar to that on Arabidopsis. *Pst* DC3000 is generally known to suppress SA production and the basal defense in host Arabidopsis plants by inducting biosynthesis of abscisic acid (ABA). Also, JA contribution to Pst DC3000 pathogenecity has also been demonstrated (de Torres-Zabala et al., 2007). ABA is a negative regulator of defense responses and has antagonistic effect on plant hormone-mediated defense pathways including that of ET (Ton et al., 2009). Therefore, it is highly possible that *SlCCaMK* role in defense against *Pst* DC3000 is linked to abscisic acid ABA signaling pathway. This is in accordance with the existing evidences that CCaMK is an ABA negative regulator. When overexpressed in Arabidopsis (which is asynbiotic), wheat CCaMK was found to negatively regulate ABA signaling (Yang et al., 2011) and, a rice CCaMK (OsDMI3) has recently been reported to positively regulate the ABA-induced antioxidant defense in rice through OsMPK1 (Shi et al., 2014).

Interestingly, we found that knock-down of *SlCCaMK* significantly reduces reactive oxygen species (ROS) accumulation after inoculation with *Pst* DC3000 (Figure 8), indicating that *SlCCaMK* plays a positive role in ROS accumulation. This is in correlation with the CCaMK negative regulation effect on ABA signaling and the mode of *Pst* DC3000 pathogenecity on Arabidopsis. In presence of ABA, various SNF1-RELATED PROTEIN KINASES 2 (SnRK2s) are activated. These include SnRK2.6 which phosphorylates and regulates the NADPH oxidase RbohF (Fujii, 2014); the main source of apoplastic ROS during pathogen-induced oxidative burst along with RbohD (Daudi et al., 2012). On the contrary, SnRK2s are negatively regulated by members of clade A PROTEIN PHOSPHATASE 2C (PP2C) (including ABSCISIC ACID INSENSITIVE (ABI) 1, ABI2, and HOMOLOGY TO ABI1 (HAB 1) (Fujii, 2014). It has been demonstrated that the Nod factors, located upstream to CCaMK, activate protein phospholipases C and D, and induce a rapid decline in H_2_O_2_ production, followed much later by the induction of H_2_O_2_ levels. In addition, Ca^2+^ spiking can directly regulate stomatal closure in plants (Oldroyd and Downie, 2004). To encounter these challenges, *Pst* DC3000 inhibits stomatal closure and delays ROS-mediated hypersensitive response (HR) cell death through its secreted polyketide toxin coronatine (Lee et al., 2013) in addition to ABA signaling hijacking.

Collectively, our results show that CCaMK positively regulates disease resistance in tomato to various pathogens including *Sclerotinia sclerotiorum* and *Pseudomonas syringae* pv. *tomato* DC3000 and promotes H_2_O_2_ accumulation.

## Conclusions

The CCaMK genes encoding full-length protein sequences were identified in additional 18 plant species. Our data confirmed the evolutionary loss of CCaMKs in cruciferous species and their absence in green algae, indicating their CCaMK prime function in rhizobial and mycorrhizal symbioses. Though it is not clear whether some differences observed in the first introns and the corresponding encoded amino acids are the basis for response specificity observed, plant CCaMKs are less diversified and structurally conserved at both gene and protein levels, demonstrating high functional conservation of the ancestral CCaMK gene during the course of evolution. Phylogenetic analysis revealed that plant CCaMK lineage is likely to have diverged from an ancestral CDPK gene in the earliest nonvascular land plant species. Functional analyses demonstrated that the tomato *CCaMK* gene was widely and differently responsive to diverse pathogenic stimuli, and positively regulates disease resistance in tomato to various pathogens including *Sclerotinia sclerotiorum* and *Pseudomonas syringae* pv. *tomato* DC3000 and promotes H_2_O_2_ accumulation. *SlCCaMK* is the first *CCaMK* gene proved to function in plant disease resistance.

## Author contributions

JW, JM, and YX conducted the bioinformatics and phylogenetic analyses. JW carried out the gene expression, ROS detection and VIGS analysis. JW and YX designed and analyzed all statistical data. XC conceived of the study, and participated in its design and coordination. XC, JW, and JM prepared the manuscript.

### Conflict of interest statement

The authors declare that the research was conducted in the absence of any commercial or financial relationships that could be construed as a potential conflict of interest.
