# Supplementary material for: Phylogeny of Plant Calcium and Calmodulin-Dependent Protein Kinases (CCaMKs) and Functional Analyses of Tomato CCaMK in Disease Resistance
Source: Front Plant Sci. 2015 Dec 8;6:1075. doi: 10.3389/fpls.2015.01075 (PMC4672059; doi:10.3389/fpls.2015.01075)
Supplement: Supplementary file 1 [file Table1.PDF]

Supplementary Material

Phylogeny of plant calcium and calmodulin-dependent protein kinases (CCaMKs) and functional analyses of tomato CCaMK in disease resistance

Ji-Peng Wang[1†], You-Ping Xu[2†], Jean-Pierre Munyampundu[1], and Xin-Zhong Cai[1,3*]

[1] Institute of Biotechnology, College of Agriculture and Biotechnology, Zhejiang University, Hangzhou, China

[2] Centre of Analysis and Measurement, Zhejiang University, Hangzhou, China

[3] State Key Laboratory of Rice Biology, Zhejiang University, Hangzhou, China.

[†] These authors have contributed equally to this work.

[*]**Correspondence:** Xin-Zhong Cai, Institute of Biotechnology, College of Agriculture and Biotechnology, Zhejiang University, 866 Yu Hang Tang Road, Hangzhou 310058, China; E-mail: xzhcai@zju.edu.cn

Supplementary Table

Table S1 Full-length CCaMKs, CDPKs and CRKs used for phylogenetic tree construction

Gene type	Species (Order, Family)	Gene	Accession number (Database)	Number of EF-hand motifs
CCaMK	**Liverworts**			
	Haplomitrium gibbsiae (Haplomitriales, Haplomitriaceae)	HgCCaMK	FJ913229 (GenBank)	3
	Treubia lacunose (Treubiales, Treubiaceae)	TlCCaMK	FJ913230 (GenBank)	3
	Dumortiera hirsuta (Marchantiales, Wiesnerellaceae)	DhCCaMK	FJ913231 (GenBank)	3
	Pellia epiphylla (Metzgeriales, Pelliaceae)	PeCCaMK	FJ913232 (GenBank)	3
	**Mosses**			
	Polytrichum juniperinum (Polytrichales, Polytrichaceae)	PjCCaMK	FJ913236 (GenBank)	3
	Physcomitrella patens (Funariales, Funariaceae)	PpCCaMK1	AY155462 (GenBank) / Phpat.021G054100 (Phytozome)	3
		PpCCaMK2	Phpat.019G070100 (Phytozome)	3
	**Hornworts**			
	Phaeoceros laevis (Notothyladales, Notothyladaceae)	PlCCaMK	FJ913240 (GenBank)	3
	Nothoceros aenigmaticus (Dendrocerotales, Dendrocerotaceae)	NaCCaMK	FJ913241 (GenBank)	3
	**Lycophyte**			
	Selaginella moellendorffii (Selaginellales, Selaginellaceae)	SmCCaMK	82473 (Phytozome)	3
	**Monocots**			

Lilium longiflorum (Liliales, Liliaceae)	LlCCaMK	U24188 (GenBank)	3
Oryza sativa (Poales, Poaceae)	OsCCaMK	AC097175.2 (GenBank)	3
Zea mays (Poales, Poaceae)	ZmCCaMK	DQ403196 (GenBank)	3
Setaria italica (Poales, Poaceae)	SiCCaMK	Si021787m (Phytozome)	3
Triticum aestivum (Poales, Poaceae)	TaCCaMK	HM595635 (GenBank)	3
Brachypodium distachyon (Poales, Poaceae)	BdCCaMK	Bradi2g21790.1 (Phytozome)	3
Panicum virgatum (Poales, Poaceae)	PvCCaMK1	Pavir.J01749.1 (Phytozome)	3
	PvCCaMK2	Pavir.Ab00605.1 (Phytozome)	3
Sorghum bicolor (Poales, Poaceae)	SbCCaMK	Sobic.010G260600 (Phytozome)	3
**Dicots**			
Aquilegia coerulea (Ranunculales, Ranunculaceae)	AcCCaMK	Aquca_093_00002 (Phytozome)	3
Mimulus guttatus (Lamiales, Phrymaceae)	MgCCaMK	Migut.N01872 (Phytozome)	3
Solanum lycopersicum (Solanales, Solanaceae)	SlCCaMK	Solyc01g096820.2.1 (Phytozome)	3
Solanum tuberosum (Solanales, Solanaceae)	StCCaMK	PGSC0003DMT400070801 (Phytozome)	3
Nicotiana tabacum (Solanales, Solanaceae)	NtCCaMK1	U38446 (GenBank)	3
	NtCCaMK2	U70923 (GenBank)	3
Petunia hybrida (Solanales, Solanaceae)	PhCCaMK	EF592572 (GenBank)	3
Vitis vinifera (Vitales, Vitaceae)	VvCCaMK	GSVIVT01027353001 (Phytozome)	3
Eucalyptus grandis (Myrtales, Myrtaceae)	EgCCaMK	Eucgr.G02633.1 (Phytozome)	3
Populus trichocarpa (Malpighiales, Salicaceae)	PtCCaMK1	Potri.008G011400.1 (Phytozome)	2

	PtCCaMK2	Potri.010G247400.1 (Phytozome)	3
Salix purpurea (Malpighiales, Salicaceae)	SpCCaMK	SapurV1A.0036s0860.1 (Phytozome)	3
Linum usitatissimum (Malpighiales, Linaceae)	LuCCaMK1	Lus10033400 (Phytozome)	3
	LuCCaMK2	Lus10034860 (Phytozome)	3
Manihot esculenta (Malpighiales, Euphorbiaceae)	MeCCaMK	cassava4.1_026542m (Phytozome)	3
Ricinus communis (Malpighiales, Euphorbiaceae)	RcCCaMK	30226.m002047 (Phytozome)	3
Citrus sinensis (Sapindales, Rutaceae)	CsCCaMK	orange1.1g009980m (Phytozome)	3
Citrus clementina (Sapindales, Rutaceae)	CcCCaMK	Ciclev10000859m (Phytozome)	3
Gossypium raimondii (Malvales, Malvaceae)	GrCCaMK	Gorai.N006600.1 (Phytozome)	3
Theobroma cacao (Malvales, Malvaceae)	TcCCaMK	Thecc1EG044590t1 (Phytozome)	3
Malus domestica (Rosales, Rosaceae)	MdCCaMK	MDP0000143220 (Phytozome)	3
Fragaria vesca (Rosales, Rosaceae)	FvCCaMK	XP_004300049 (NCBI)	3
Prunus persica (Rosales, Rosaceae)	PpeCCaMK	ppa004207m (Phytozome)	3
Cucumis sativus (Cucurbitales, Cucurbitaceae)	CsaCCaMK	Cucsa.364320.1 (Phytozome)	3
Glycine max (Fabales, Fabaceae)	GmCCaMK1	Glyma.08G227200.1 (Phytozome)	3
	GmCCaMK2	Glyma.15G222300.1 (Phytozome)	3
Medicago truncatula (Fabales, Fabaceae)	MtCCaMK	AY502066 (GenBank)	3
Phaseolus vulgaris (Fabales, Fabaceae)	PvuCCaMK	Phvul.011G186900 (Phytozome)	3
Lotus japonicus (Fabales, Fabaceae)	LjCCaMK	AM230792 (GenBank)	3
Sesbania rostrata (Fabales, Fabaceae)	SrCCaMK	EU622875 (GenBank)	3

	Arachis hypogaea (Fabales, Fabaceae)	AhCCaMK	EU395429 (GenBank)	3
	Pisum sativum (Fabales, Fabaceae)	PsCCaMK	AY502067 (GenBank)	3
CDPK	**Apicomplexan**			
	Toxoplasma gondii (Eucoccidiorida, Sarcocystidae)	TgCDPK1	TGME49_301440 (ToxoDB)	4
	Cryptosporidium parvum (Eucoccidiorida, Cryptosporidiidae)	CpCDPK1	cgd3_920 (CryptoDB)	4
	Plasmodium falciparum (Haemosporida, Plasmodiidae)	PfCDPK3	PF3D7_0310100 (PlasmoDB)	4
	**Dicot**			
	Arabidopsis thaliana (Brassicales, Brassicaceae)	AtCDPK1	At5g04870 (TAIR)	4
		AtCDPK3	At4g23650 (TAIR)	4
		AtCDPK8	At5g19450 (TAIR)	4
		AtCDPK10	At1g18890 (TAIR)	4
		AtCDPK12	At5g23580 (TAIR)	4
		AtCDPK13	At3g51850 (TAIR)	4
		AtCDPK16	At2g17890 (TAIR)	4
		AtCDPK17	At5g12180 (TAIR)	4
		AtCDPK18	At4g36070 (TAIR)	4
		AtCDPK20	At2g38910 (TAIR)	4
		AtCDPK28	At5g66210 (TAIR)	4
		AtCDPK29	At1g76040 (TAIR)	4
CRK	**Dicot**			

Arabidopsis thaliana (Brassicales, Brassicaceae)	AtCRK1	At2g41140 (TAIR)	0
	AtCRK2	At3g19100 (TAIR)	0
	AtCRK3	At2g46700 (TAIR)	0
	AtCRK4	At5g24430 (TAIR)	0
	AtCRK5	At3g50530 (TAIR)	0
	AtCRK6	At3g49370 (TAIR)	0
