# Supplementary material for: Phylogeny of Plant Calcium and Calmodulin-Dependent Protein Kinases (CCaMKs) and Functional Analyses of Tomato CCaMK in Disease Resistance
Source: Front Plant Sci. 2015 Dec 8;6:1075. doi: 10.3389/fpls.2015.01075 (PMC4672059; doi:10.3389/fpls.2015.01075)
Supplement: Supplementary file 2 [file Image1.PDF]

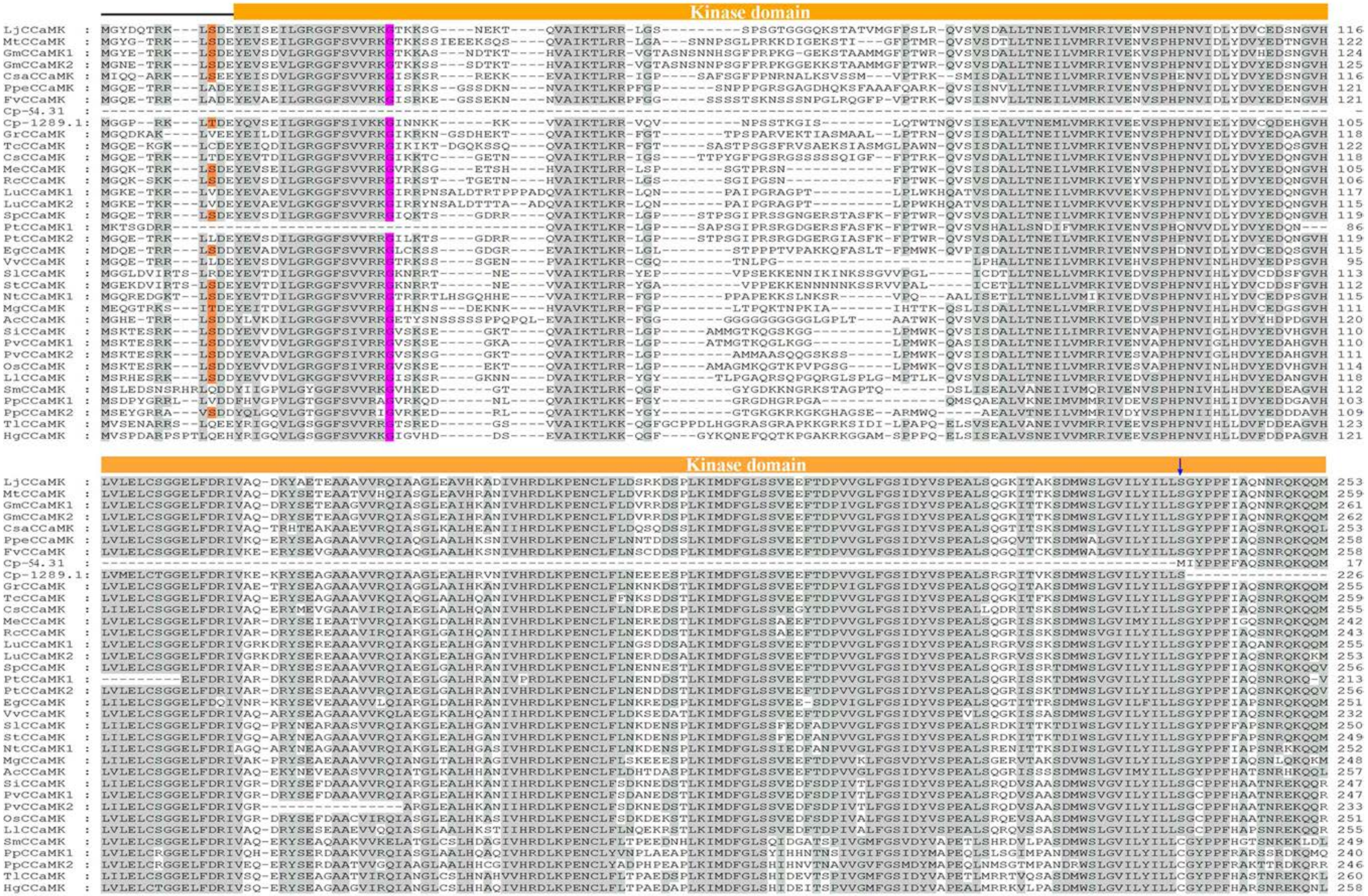
Kinase domain
Kinase domain

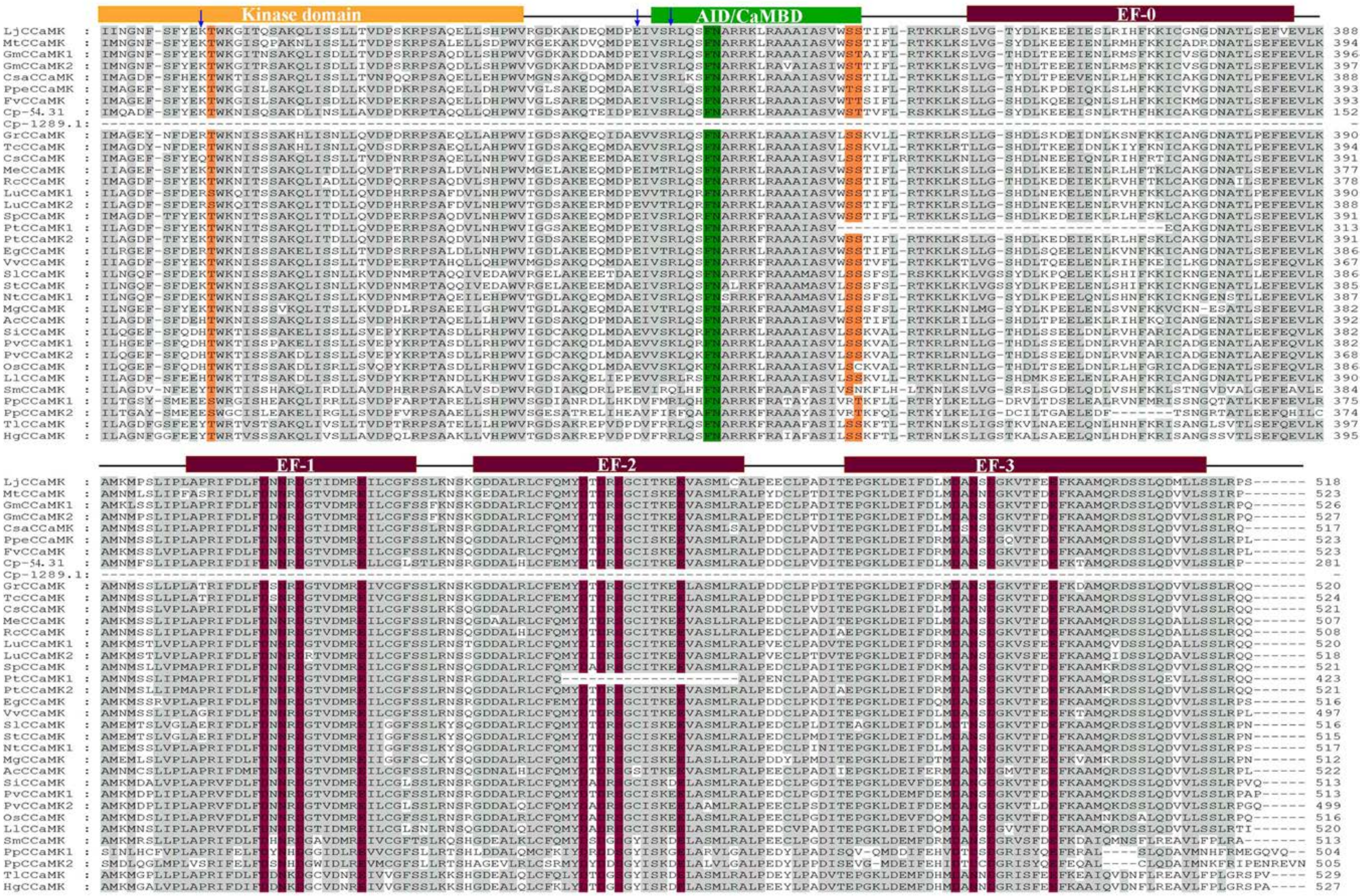

Supplementary Figure 1: Protein sequence alignments of CCaMK representatives from various species. All plant CCaMKs are conserved except N-terminal of kinase domain. Most of CCaMK regulatory residues are also conserved except the

autophosphorylation site corresponding to S9 in *M. truncatula* CCaMK (MtCCaMK). *C. papaya* incomplete sequences, evm.TU.supercontig_1289.1 (Cp-1289.1) with only part of the kinase domain and evm.TU.supercontig_54.31 (Cp-54.31) containing incomplete C-terminal kinase domain and the three complete EF-hand motifs, were aligned with full-length CCaMKs from different phylogenetically unrelated plant species. The kinase domain, overlapping autoinhibitory/Calmodulin binding domain (AID/CaMBD), and two EF-hand domains (two pairs, EF-0; EF-1 and EF-2; EF-3) of *L. japonicus* CCaMK (LjCCaMK) domains are shown in different colors above the alignments. The residues in orange, green and purple background shading indicate CCaMK autophosphorylable sites, CaM-binding site, Ca^{2+} binding sites in the three EF-hands, respectively. EF-0 represents the CCaMK degenerated EF-hand motif. The position of the residue G30, which is required for CCaMK kinase activity of LjCCaMK, is shown in pink background, while residues participating in hydrogen-bond engaged by autophoshorylation site T265 in LjCCaMK are indicated by blue arrows. The domains were drawn based on the description by Shimoda et al. (2012) and prediction using Prosite programs.
